# Functional hypothalamic amenorrhea: Impact on bone and neuropsychiatric outcomes

**DOI:** 10.3389/fendo.2022.953180

**Published:** 2022-07-22

**Authors:** Clarissa Carvalho Pedreira, Jacqueline Maya, Madhusmita Misra

**Affiliations:** ^1^ Division of Pediatric Endocrinology, Massachusetts General Hospital and Harvard Medical School, Boston, MA, United States; ^2^ Neuroendocrine Unit, Center for Endocrinology and Diabetes of Bahia State, Salvador, Brazil; ^3^ Neuroendocrine Unit, Massachusetts General Hospital and Harvard Medical School, Boston, MA, United States

**Keywords:** functional hypothalamic amenorrhea, estrogen deficiency, bone health, anxiety, depression, adolescent

## Abstract

Functional hypothalamic amenorrhea is a state of reversible hypogonadism common in adolescents and young women that can be triggered by energy deficit or emotional stress or a combination of these factors. Energy deficit may be a consequence of (i) reduced caloric intake, as seen in patients with eating disorders, such as anorexia nervosa, or (ii) excessive exercise, when caloric intake is insufficient to meet the needs of energy expenditure. In these conditions of energy deficit, suppression of the hypothalamic secretion of gonadotrophin-releasing hormone (with resulting hypoestrogenism) as well as other changes in hypothalamic-pituitary function may occur as an adaptive response to limited energy availability. Many of these adaptive changes, however, are deleterious to reproductive, skeletal, and neuropsychiatric health. Particularly, normoestrogenemia is critical for normal bone accrual during adolescence, and hypoestrogenemia during this time may lead to deficits in peak bone mass acquisition with longstanding effects on skeletal health. The adolescent years are also a time of neurological changes that impact cognitive function, and anxiety and depression present more frequently during this time. Normal estrogen status is essential for optimal cognitive function (particularly verbal memory and executive function) and may impact emotion and mood. Early recognition of women at high risk of developing hypothalamic amenorrhea and its timely management with a multidisciplinary team are crucial to prevent the severe and long-term effects of this condition.

## Introduction

Functional hypothalamic amenorrhea (FHA) is a condition characterized by irregular or absent menses due to suppression of the hypothalamic–pituitary–ovarian (HPO) axis, and the condition is termed ‘functional’ because no anatomical or organic disease is identified (1). Abnormalities in gonadotropin-releasing hormone (GnRH) secretion (2, 3) result in hypogonadotropic hypogonadism with impaired luteinizing hormone (LH) pulsatile secretion (4–8), and insufficient LH and follicle-stimulating hormone (FSH) concentrations (5, 9) to maintain full folliculogenesis and therefore ovulatory function, and the condition is also referred to as functional hypogonadotropic hypogonadism. FHA is a common cause of secondary amenorrhea in young premenopausal women and results in severe hypoestrogenism. According to the American Society of Reproductive Medicine, FHA is responsible for 20–35% of secondary amenorrhea (10). It typically occurs in the setting of low body weight, such as anorexia nervosa (AN), excessive exercise (exercise induced or athletic amenorrhea), stress, or a combination of these factors (5). AN is a chronic, relapsing disease defined in the Diagnostic and Statistical Manual -5 (DSM-5) as a state of low body weight in the setting of altered body image and fear of weight gain (11). The Female Athlete Triad (TRIAD) refers to the triad of low energy availability, menstrual dysfunction, and low bone mineral density (BMD) (12–14). Energy deficiency, from either a frank deficit in caloric intake or relative to excessive exercise, leads to hormonal adaptations that aim to optimize energy availability and prioritize this for body functions essential for survival.

Adolescents and young women with FHA typically present with amenorrhea of 6 months’ duration or longer (15). However, menstrual status can vary ranging from subclinical menstrual dysfunction (including a shortened luteal phase or anovulatory cycles) to frank oligo-amenorrhea. In adolescents, this condition may be difficult to distinguish from immaturity of the hypothalamic–pituitary–ovarian axis during the early postmenarchal years. However, several reports now indicate that even during the initial postmenarchal years menstrual cycles in adolescents typically are no longer than 45 days, thus irregular or absent menses are concerning (16–18).

The process by which GnRH is suppressed in FHA is multifactorial, as there are many inhibitory and stimulatory neuromodulatory signals that impair GnRH pulsatility. Kisspeptin plays a fundamental role in regulating reproductive function. In the human brain, kisspeptin neurons are found in the hypothalamus, basal ganglia, and periventricular region (19, 20). Kisspeptin has been implicated as the common intermediate signaling factor modulating GnRH activity, acting downstream of leptin and other neuromodulatory systems (21). Studies have demonstrated reduced kisspeptin secretion in conditions of energy deficit in rodents (22). This may be mediated by reductions in levels of hormones such as leptin, insulin, and insulin-like growth factor-1 (IGF-1), and increases in hormones such as ghrelin, cortisol, and adiponectin (23–26).

There may also be a genetic predisposition for the development of FHA. One small study identified heterozygous mutations in the fibroblast growth factor receptor 1 gene *FGFR*, the prokineticin receptor 2 gene *PROKR2*, the hypothalamic gonadotrophin-releasing hormone receptor gene *GNRHR*, and the Kallmann syndrome 1 sequence gene *KAL1* (27) in patients with FHA, suggesting an increased vulnerability to develop hypothalamic amenorrhea. These mutations were not found in healthy controls. However, these findings have not been replicated and more data are necessary to determine whether these findings hold in a larger sample.

The prolonged hypoestrogenemia in FHA has profound effects on many body systems including metabolic, skeletal, neuropsychiatric, and reproductive systems. Estrogen plays an important role in bone health and therefore estrogen deficiency can impact bone mass deleteriously (4, 28). This is particularly important during adolescence, a critical period for bone mass accrual, and lack of estrogen during this time can lead to decreased BMD and increased fracture risk, both immediate and in the long-term. Another area of concern is the impact of prolonged hypogonadism and changes in hormones such as cortisol, leptin, and peptide YY (PYY) on neurocognitive status, emotion, and mood (29–32), thus posing additional challenges at an age when emotional lability is already common. The adolescent years are very important for optimal development of neuropsychiatric function and normoestrogenemia may be essential in this context.

Many young women are not aware of these long-term effects of FHA; thus, it is important to identify these women early, and address with them the importance of adequate energy availability and resumption of menses. Treatment includes correction of the energy deficit state to improve GnRH pulsatility and restore normal functioning of the HPO axis.

## Impact of conditions associated with functional hypothalamic hypogonadism on bone

In situations of prolonged hypoestrogenism, as seen in states of functional hypothalamic (or hypogonadotropic) hypogonadism, changes are noted in areal BMD (aBMD), bone microarchitecture and strength estimates, associated with an increased risk of fractures in these individuals.

### Anorexia nervosa and bone

Bone health has been extensively reviewed among adolescents with AN. Compared to controls, adolescent and young adult women with AN have low aBMD, which is driven independently by both low body weight (percent expected body weight for height, EBW-Ht, ≤ 80 or 90%) and amenorrhea (33), and alterations in hormones such as IGF-1, the gonadal steroids, cortisol, leptin, insulin, adiponectin, PYY and oxytocin. Older studies indicate that as many as 67% of these individuals may have an aBMD Z-score < -2 (34) while more recent findings (given earlier diagnosis of AN) report that ~ 52% have an aBMD Z-score < -1 at one or more sites, with trabecular bone (spine) being commonly affected (35). Of particular concern, in AN, bone accrual is stalled during adolescence, a vital period that determines long term bone health and fracture risk (36, 37). BMD improves with weight gain and menstrual recovery. However, it is uncertain whether full catch-up occurs and whether this is sufficient to ensure optimal long-term bone health (37).

**Figure 1:**
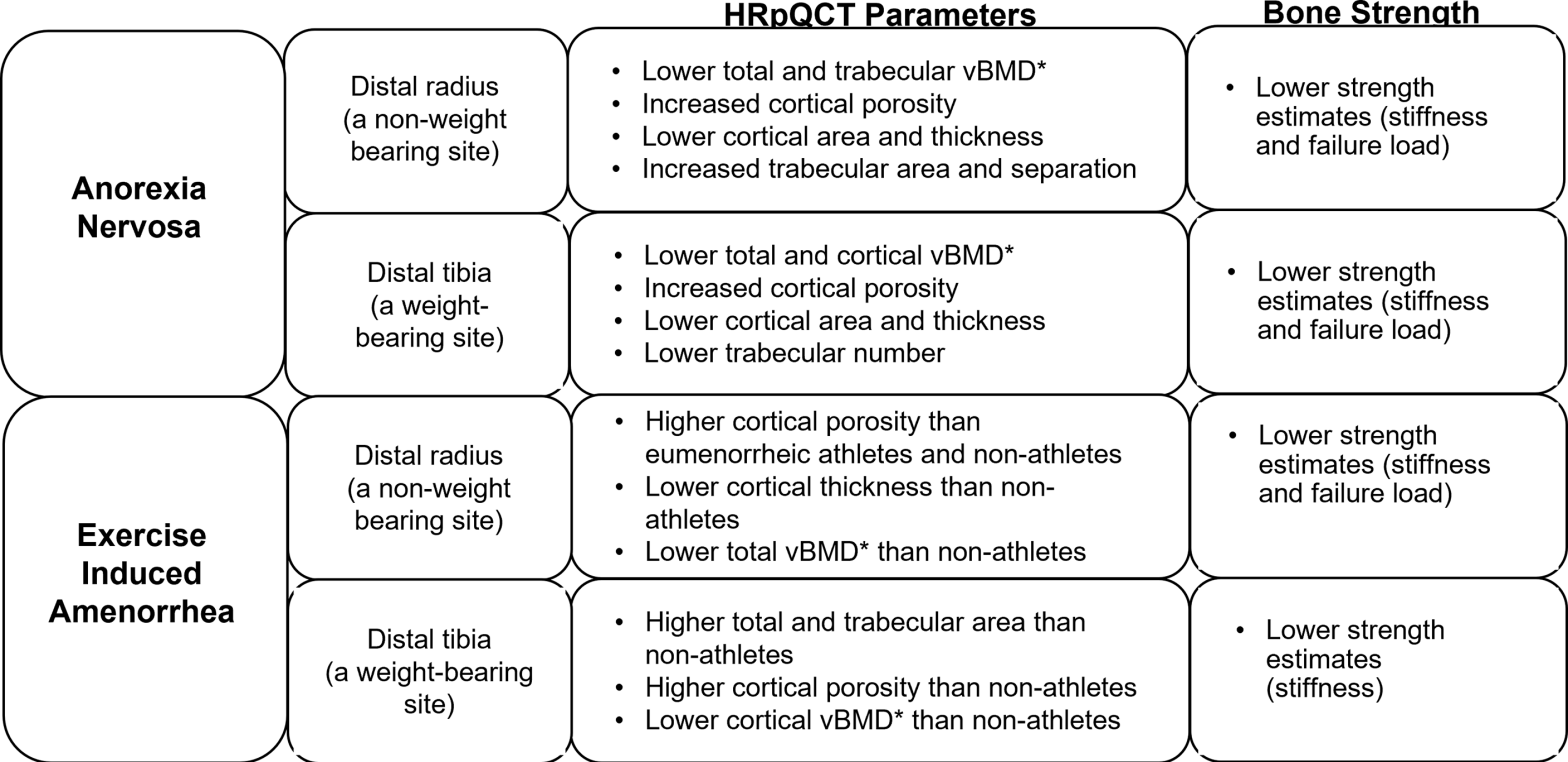
Impact of functional hypothalamic amenorrhea on bone parameters as assessed by high-resolution peripheral quantitative computed tomography (HRpQCT) and microfinite element analysis (μFEA). HRpQCT was used to assess volumetric bone mineral density (vBMD)*, bone geometry and structure, and μFEA to assess bone strength estimates at the distal radius and tibia.

Furthermore, studies using high-resolution peripheral quantitative computed tomography (HRpQCT) and microfinite element analysis (µFEA) (**Figure 1**) have demonstrated changes in cortical and trabecular volumetric BMD (vBMD), bone geometry and microarchitecture, and bone strength estimates in AN. At the distal radius (a non-weight bearing site), compared to controls, adolescents and young women with AN have lower total and trabecular vBMD, increased cortical porosity, lower cortical area and thickness, increased trabecular area and separation, and lower strength estimates (stiffness and failure load) (38). At the distal tibia (a weight-bearing site) adolescents and young adult women with AN, compared with controls, have lower total and cortical vBMD, increased cortical porosity, lower cortical area and thickness, lower trabecular number and lower stiffness and failure load (39). Another study showed, lower trabecular bone volume fraction (BV/TV) and trabecular thickness and higher trabecular separation in girls with AN using flat panel volume computed tomography (CT) when compared with normal-weight controls (40). IGF-1, leptin and androgen levels predict bone microarchitecture in adult women with AN, with lower levels appearing to have deleterious effects (41). Marrow adipose tissue (MAT) measured by 1H-magnetic resonance spectroscopy (1H-MRS) is higher in AN than controls, indicating increased differentiation of the common mesenchymal progenitor stem cell in marrow along the adipocyte rather than the osteoblast lineage, and higher marrow adipose tissue is related to lower bone strength estimates in young women with this condition (39).

These bone changes translate to higher fracture risk in adolescents and adult women with AN compared with controls that persists over time (42–44). One study reported no difference across groups in the site of fracture (upper extremity, lower extremity or non-extremity); the AN group just had many more fractures across all sites than the control group (44).

### Exercise induced amenorrhea and bone

Similarly, in oligomenorrheic athletes, bone density, bone microarchitecture and strength are altered, a consequence of both low energy availability (from insufficient caloric intake and increased metabolic demands) and hypoestrogenism, as well as alterations in hormones such as IGF-1, other gonadal steroids, cortisol, and other metabolically regulated hormones. Oligoamenorrheic athletes have lower spine, hip and whole body aBMD than eumenorrheic athletes (45, 46). As in AN, there is concern that catch-up may be insufficient even after menses resume given the narrow window for optimizing bone accrual during and after puberty (47, 48).

However, using only BMD to assess bone health may be insufficient (14). In oligomenorrheic athletes, HRpQCT has been used to study changes in bone geometry and microarchitecture, and findings differ at non-weight bearing compared with weight bearing sites (**Figure 1**). At non-weight bearing sites such the distal radius, oligomenorrheic athletes have higher cortical porosity and lower strength estimates than eumenorrheic athletes, and higher cortical porosity with lower cortical thickness and total vBMD than non-athletes (46). At weight bearing sites such as the tibia, oligomenorrheic athletes have higher total and trabecular area, higher cortical porosity and lower cortical vBMD than non-athletes, and lower strength estimates than both eumenorrheic athletes and non-athletes (46, 49). Further, in one study, trabecular number was lower and separation higher in oligomenorrheic athletes compared to eumenorrheic athletes and non-athletes at the tibia (50). More recently, a study assessed the effects of energy deficiency (defined as energy intake below 45 kcal/kg fat-free mass/day) in long-distance triathletes without hypoestrogenism compared to non-athletes and found that several bone parameters (total and trabecular area, trabecular vBMD and trabecular microstructure) were better in athletes than non-athletes (consistent with the adaptive effects of bone loading), but inferior in athletes with low energy availability compared to those with adequate energy availability (51).

Fractures, particularly stress fractures, are more prevalent among oligo-amenorrheic athletes than eumenorrheic athletes and non-athletes (46). A history of two or more fractures in oligo-amenorrheic athletes has been associated with lower spine and whole-body BMD Z-scores, lower radial cross-sectional area, trabecular vBMD and strength estimates, and lower tibial strength estimates compared to oligoamenorrheic athletes with less than 2 fractures (46). Thus, the protective mechanism bestowed by mechanical loading on athletes is deficient in a state of hypoestrogenism.

### Anorexia nervosa versus female athlete triad

Bone health and fracture risk are affected differently in adolescents and young adult women with AN and oligo-amenorrheic athletes (52). In AN, whole body less head (WBLH) and hip aBMD Z-scores, and several measures at the weight-bearing tibia (total vBMD, cortical area and thickness, trabecular number, and estimated strength) were lower than in oligo-amenorrheic athletes and controls in one study (52). In contrast, both AN and oligo-amenorrheic athletes had lower spine aBMD Z-scores, lower radius total, cortical and trabecular vBMD, cortical area, cortical thickness, and estimated strength, and lower tibial cortical vBMD than controls (52). Although fracture risk was higher in women with AN and oligo-amenorrheic athletes, the fracture type varied with nonstress fractures being more common in AN and stress fractures being more common in oligo-amenorrheic athletes.

## Determinants of bone health in functional hypothalamic hypogonadism

**Figure 2:**
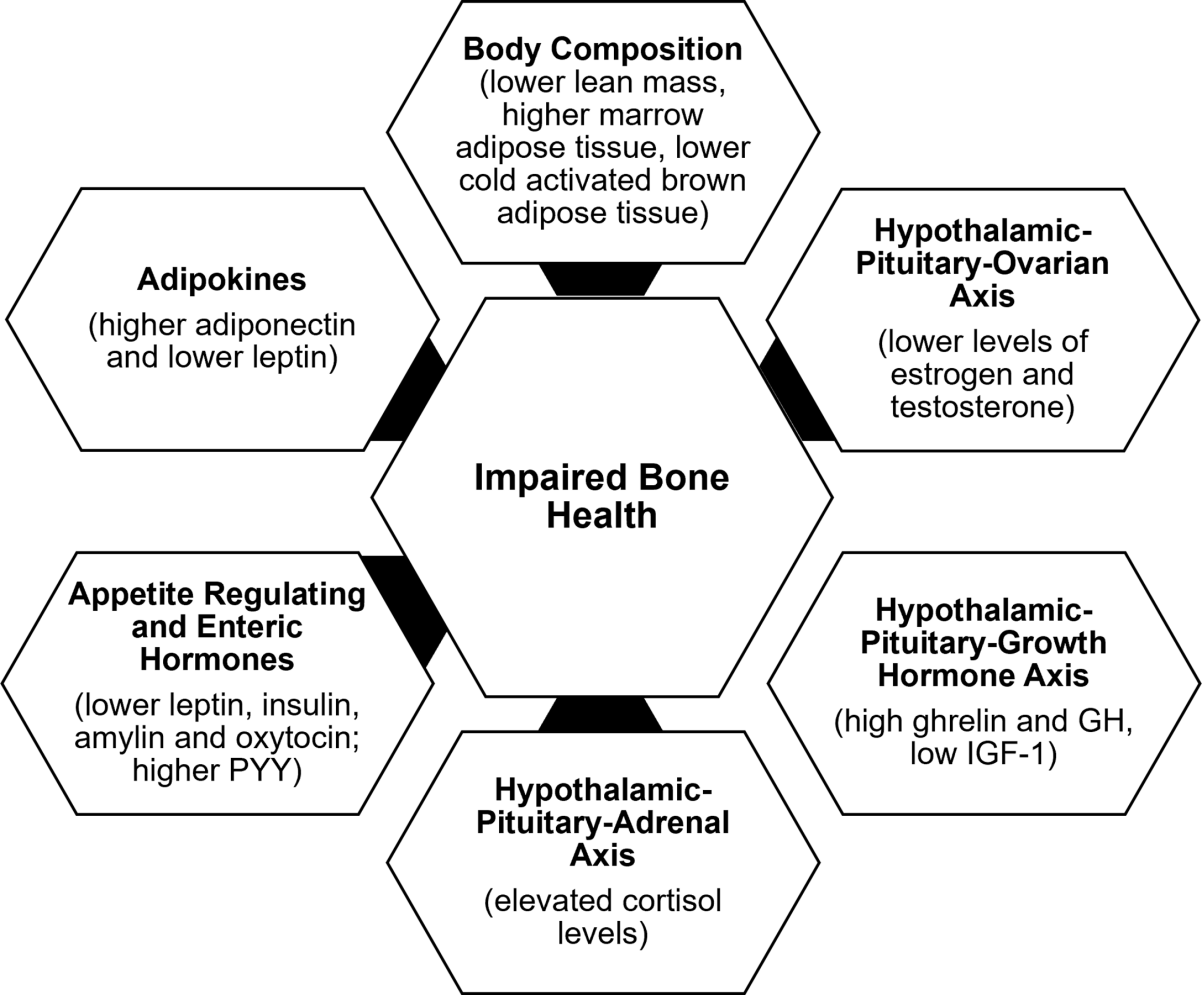
Factors contributing to impaired bone health in functional hypothalamic amenorrhera.

Determinants of bone outcome in these patients include changes in body composition, alterations in the HPO axis, growth homone-IGF-1 axis, hypothalamic-pituitary-adrenal axis, and appetite regulating and other hormones, consequent to the low energy availability state (**Figure 2**).

### Body composition

In addition to the lower body mass index (BMI), changes in body composition in the setting of energy deficiency impact bone health. Overall, restrictive caloric intake in AN leads to lower fat and lean mass and lower resting energy expenditure (53). A compensatory increase in cortisol concentrations occurs in energy deficient states such as AN and exercise induced amenorrhea, and in AN, higher cortisol concentrations are associated with lower extremity lean mass (54). Lower lean mass is an independent predictor of BMD at almost every site, consistent with the pull of muscle on bone being osteogenic (37). With weight regain, increases in lean mass are associated with increases in BMD (36). Further, marrow adipose tissue (known to reduce biomechanical strength) is higher in AN than controls and in oligo-amenorrheic athletes than in eumenorrheic athletes and is associated with lower BMD and lower strength estimates (55, 56). In contrast, cold-activated brown adipose tissue is lower in AN. Some believe that brown adipose tissue plays a role in the differentiation of a common marrow progenitor mesenchymal stem cell preferentially into osteoblasts instead of adipocytes (57). This would explain a direct association of lower brown adipose tissue with lower BMD and an inverse relationship with levels of pre-adipocyte factor-1 (pref-1), a hormone that inhibits differentiation of this mesenchymal stem cell along the osteoblast pathway (57).

### Hypothalamic-pituitary-ovarian axis

The gonadal steroids, including estrogen, testosterone and dehydroepiandrosterone (DHEA) (an ovarian and adrenal androgen and estrogen precursor), have important effects on bone (28).The estrogens (estradiol and estrone) inhibit osteoclastic bone resorption by increasing osteoprotegerin and decreasing receptor activator of nuclear factor kappa-B ligand (RANKL) secretion by osteoblasts (28). They may also increase bone formation by inhibiting secretion of sclerostin and pref-1, both of which otherwise inhibit osteoblast differentiation. Effects of testosterone on bone are mediated *via* its aromatization to estrogen; however, it also has direct osteoanabolic and anti-resorptive effects. DHEA is weakly bone anabolic, and also anti-resorptive through its aromatization to estrogen. Levels of estrogen and testosterone are lower in AN and in oligo-amenorrheic athletes compared with controls (36, 50). The duration of amenorrhea, consistent with the duration of hypogonadism, and menarchal age predict the extent of bone health impairment.

### Hypothalamic-pituitary-growth hormone axis

Energy deficiency results in a state of growth hormone (GH) resistance with elevated GH concentrations and low IGF-1 levels in AN vs. controls (58), and in amenorrheic athletes vs. non-athletes (45), consistent with a hepatic resistance to GH, likely mediated by a downregulation of the GH receptor in end organs (as indicated by lower levels of GH binding protein) (59) and elevated fibroblast growth factor (FGF)-21 concentrations (60). This state of GH resistance is commonly seen in conditions of undernutrition. In athletes, lower IGF-1 levels have been associated with higher intensity of training (61, 62). GH stimulates osteoblast precursors and mature osteoblasts both directly and indirectly through the action of IGF-1 (63). While higher GH concentrations are associated with higher levels of bone turnover markers in healthy normal-weight controls, this association is not evident in AN (despite higher GH concentrations), indicative of a resistance to GH at the level of bone (in addition to the liver) (58). A study using supraphysiologic doses of recombinant human GH demonstrated a decrease in fat mass in women with AN (consistent with its IGF-1 independent lipolytic effects) without a corresponding increase in IGF-1 concentrations or concentrations of P1NP (a bone formation marker), further corroborating a hepatic and skeletal resistance to GH in AN (64). Further, low IGF-1 concentrations are associated with low bone density in conditions of functional hypothalamic hypogonadism (36, 45), and administration of replacement doses of recombinant human IGF-1 (rhIGF-1) has been associated with an increase in levels of bone formation markers in adolescents and adults with AN (65, 66).

### Hypothalamic- pituitary-adrenal axis

One of the neuroendocrine adaptations of FHA includes overactivity of the hypothalamic–pituitary–adrenal axis (HPA), with increased secretion of corticotropin-releasing hormone (CRH), adrenocorticotropin hormone (ACTH), cortisol, and endogenous opioids (67–71). Higher cortisol levels have also been found in the cerebrospinal fluid of women with FHA compared to eumenorrheic women (7, 72). There is a tight link between activation of the HPA axis and reduction in GnRH drive in those with FHA (5, 70, 73, 74), such that an increase in CRH suppresses GnRH pulsatility (7). Further, cortisol inhibits kisspeptin release (75, 76), and its elevation contributes to the cascade of impairment in GnRH release, with higher cortisol concentrations being associated with lower secretion of LH (70). One study has shown that in AN, adolescents with the lowest BMI, fat mass, fasting glucose and insulin levels (thus with the lowest energy availability) have the highest cortisol concentrations, suggesting that the increase in cortisol levels is an adaptative mechanism, likely to maintain euglycemia in a state of low availability given its gluconeogenic effects (71).

These relatively high cortisol levels may have immediate and long-term effects on bone health in patients with FHA. The deleterious effects of hypercortisolemia on bone are mediated by many different mechanisms including reduced osteoblastic activity, increased osteoclastic activity, inhibition of intestinal calcium absorption, impaired renal handling of calcium, and reduced secretion of GH and IGF-1 (77). Higher cortisol levels predict lower percent extremity lean mass (54) and lower bone density in FHA (30, 71). As previously discussed, lean body mass is an important determinant of bone density (35, 78). High cortisol levels in women with AN are inversely correlated with markers of bone turnover and may contribute to low BMD through suppression of bone formation (71, 79).

### Insulin, enteric peptides and adipokines

Appetite regulating hormones (such as leptin, insulin, PYY, and oxytocin, which are anorexigenic, and ghrelin, which is orexigenic) as well as adipokines, such as adiponectin, are modulators of energy availability and play a critical role in the regulation of hypothalamic dysfunction and in bone metabolism in FHA (80, 81). Leptin and insulin stimulate kisspeptin release, while ghrelin and adiponectin inhibit its release (25, 75, 76, 82, 83). Similarly, PYY can modulate reproductive function (84, 85). Women with FHA have lower leptin (6, 8, 9, 86–90), insulin (9, 86, 87, 91), and oxytocin levels (92), and higher ghrelin (8, 90, 93–95), PYY (94, 96, 97), and adiponectin (91, 98) levels than controls. In FHA, many of these hormonal alterations have been associated with suppression of the HPO axis (25, 80, 81, 99, 100).

All these hormonal alterations contribute to hypogonadism in FHA and consequently to low bone mass. In addition, these hormones have direct effects on bone. Leptin is osteoanabolic and antiresorptive (101–103), and insulin, amylin and ghrelin also have bone anabolic effects. Lower levels of leptin, insulin and amylin correlate with lower BMD and impaired bone microstructure in those with AN (41, 91, 104). Ghrelin levels correlate with bone endpoints in healthy normal-weight controls, but not in girls with AN, consistent with a ghrelin resistant state (105). PYY inhibits osteoblastic activity (106), and in AN, high PYY levels are associated with lower BMD in adults, and with lower levels of bone turnover markers in adolescents (96, 107). Similarly, higher PYY levels are associated with lower levels of bone formation markers and lower BMD in adolescent athletes and non-athletes (97). Oxytocin is now known to be bone anabolic, and lower oxytocin concentrations in AN have been associated with lower bone density (108). Adiponectin receptors are expressed on osteoblasts and osteoclasts (109, 110), and high levels of adiponectin are deleterious to bone. High adiponectin levels are associated with low BMD in healthy adults (111, 112) and in girls with AN (91).

## Treatment strategies

The first line of management of FHA is lifestyle intervention, aimed at normalization of HPO axis function and resumption of menses. The approach should be multidisciplinary and include involvement of a physician to coordinate care (preferably an eating disorder specialist), a dietician, and a psychologist or psychiatrist (particularly when there is a co-existing eating disorder), with engagement of the parents or other family members and the athletic trainer or coach (for hyperexercisers). The condition is generally reversible and resolves after restoration of energy balance and resolution of underlying emotional stress. While targeting the triggers of FHA, such as disordered eating, excessive exercise or emotional stress is the first approach, convincing patients to change long-standing behavior can be challenging.

A dietary evaluation and consequent counseling are important to optimize caloric intake (including healthy fat), and micronutrients such as calcium and vitamin D. Energy availability should meet established weight goals and other clinical criteria for athletes to continue exercising, and these athletes may need to modify their training and competition regimen if such goals are not met (46, 113). Consistent with this, sports consensus groups, including the Female Athlete Triad Coalition and International Olympic Committee, recommend that athletes with FHA undergo screening for various components of the Triad and meet certain energy availability requirements to be permitted to continue to exercise (12, 13). In a small study, three out of four amenorrheic athletes resumed menses after a 20-week program that included nutritional supplementation and one rest day per week (114).

Similarly, psychological support for treating stress and enhancing behavioral change is critical (14). Behavioral modifications can result in a reduction in cortisol levels (115) and resumption of ovarian function in some women with FHA (116). One study showed that 71% of patients recovered over a period of 7-9 years and predictive factors of recovery included lower serum cortisol concentrations and higher basal BMI (117).

There has been some debate over whether a critical increase in weight is required to resume menstrual cycles. Based on one study, a recommendation is that goal weight should be at least 2 kg higher than the weight at which point menses were lost (118). This longitudinal study involving adolescents with AN also showed that menstruation resumed at a mean body weight that was 91.6 **±** 9.1% of ideal body weight and that it could take 6-12 months or longer of being at a ‘healthy’ weight before menses resumed (118). Another study suggested that about 50% of women are expected to resume their menstrual cycle when they are at or above a BMI of 19 kg/m^2^ with ≥ 23% body fat (119). Overall, the recommendations are to regain any recent weight loss, to aim for a body weight that is at least 2 kg greater than at which menses were lost and a body weight that corresponds to greater than 90% of median BMI for age (or greater than 18.5 kg/m^2^ for adults). However, there is significant variability in the set point for menstrual recovery from one individual to another, thus establishing goal weight can be challenging. Further, if a woman continues to be amenorrheic despite being at a healthy weight for a prolonged period, it is important to consider other causes of amenorrhea such as persistent emotional stress or conditions such as polycystic ovarian syndrome.

In women, particularly athletes, who are at a ‘healthy’ weight and yet have FHA, low caloric intake may still be a contributor to the amenorrheic state, these women often have lower fat mass than eumenorrheic women, consistent with a state of energy deficit. In such women, careful assessment of caloric intake and expenditure may be necessary to demonstrate the state of low energy availability. Individualized meal plans are often helpful in such instances, and it is important to emphasize optimizing caloric intake before and after periods of intense exercise. Recommendations from the Female Athlete Triad Coalition include increasing calories through intake of food such as nuts, dried fruit, energy bars and drinks, avocado and fatty fish (12).

## Management of low bone density

**Figure 3:**
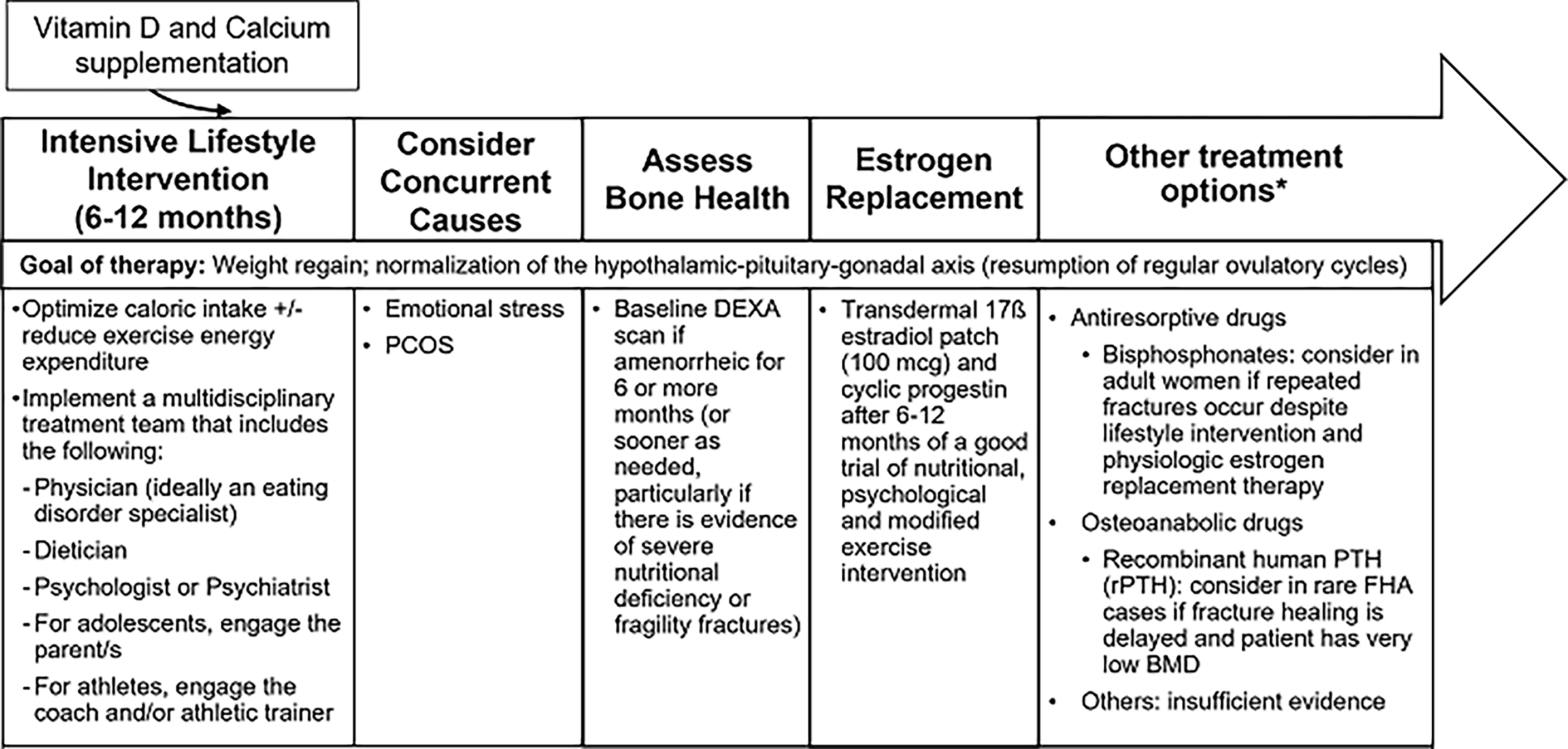
Management of Functional Hypothalamic Amenorrhea. HPO, Hypothalamic-pituitary-ovarian axis; PCOS, Polycystic ovarian syndrome; DEXA, Dual-energy X-ray absorptiometry; rPTH, Recombinant parathyroid hormone. *Treatment is similar in both adult and adolescent women except for "other treatment options" which at this time, only apply to adults.

The management of low bone density in FHA is summarized in **Figure 3**. **Table 1** summarizes interventional studies addressing bone outcomes in FHA.

**Table 1:** Interventional studies in the management of functional hypothalamic amenorrhea.

Study	Population	Intervention	Outcomes
***Oral estrogen-progestin therapy***			
Klibanski A, et al. The Journal of Clinical Endocrinology & Metabolism. 1995 (120).	48 amenorrheic women with AN mean age 23.7 years	Estrogen and progestin replacement (n=22) vs. no replacement (n=26) for a mean of 1.5 years	Intervention (estrogen and progestin) group had no significant change in BMD compared to the group that received no hormone replacement therapy. On post-hoc analysis, very low-weight women with <70% ideal body weight treated with estrogen and progestin had a 4.0% increase in mean BMD while the group that did not receive replacement treatment had a 20.1% decrease in BMD.
Warren MP, et al. Fertility and Sterility. 2003 (121).	55 dancers (n=24) with amenorrhea mean age 22 years	Individuals with amenorrhea received conjugated equine estrogen, 0.625 mg, vs. placebo for 25 days, with medroxyprogesterone acetate 10 mg, for 10 days of every month over 2 years. Both groups were compared to eumenorrheic controls.	There was no significant difference in BMD at the lumbar spine, wrist and foot in the treated or placebo group compared to the eumenorrheic dancers.BMD increased but did not normalize in 5 individuals who resumed menses.
Strokosch GR, et al. Journal of Adolescent Health. 2006 (122).	112 adolescent girls with AN or eating disorder not otherwise specified 11-17 years old	A combined oral contraceptive (COC) (norgestimate 180-250 µg and ethinyl estradiol 35 mcg) vs. placebo for 13 cycles of 28-day cycles	Significant increase in BMD at the lumbosacral spine and hip in the intervention group at week 6. No statistically significant effect on lumbosacral spine or hip BMD at the end of 13 cycles.
***Transdermal estradiol administration with cyclic progestin***			
Ho KKY, Weissberger AJ. Journal of Bone and Mineral Research. 2009 (123)	14 postmenopausal women (two groups n=7 each)	Oral (20 µg/day of ethinyl estradiol) vs. transdermal (100 µg/day of 17β-estradiol) estrogen over 2 months	Transdermal estrogen significantly increased IGF-1, procollagen III, procollagen I, osteocalcin and fasting urinary hydroyproline to creatinine ratio (UOHPr/Cr) while oral estrogen administration led to suppression of these biochemical endpoints. There was a significant association between IGF-1 elevation and changes in procollagen III, procollagen I, osteocalcin and UOHPr/Cr.
Misra M, et al. Journal of Bone and Mineral Research. 2011 (124).	110 girls with AN and 40 normal-weight controls 12-18 years	Girls with AN: Those with a bone age of ≥15 years (n=96) received 100 µg of 17β-estradiol + cyclic medroxyprogesterone acetate vs. placebo, while those with a bone age of <15 years (n=14) received incremental low-dose oral ethinyl-estradiol vs. placebo for 18 months. 40 normal-weight controls were followed without intervention for the study duration	Spine and hip BMD and BMD Z-scores improved in the group with AN that received physiologic estrogen replacement to approximate bone accrual rates observed in controls.
Ackerman KE, et al. British Journal of Sports Medicine 2019 (47)	121 normal-weight athletes with amenorrhea 14-25 years	100 µg 17β-estradiol transdermal patch + cyclic 200 mg oral micronized progesterone vs. 30 µg ethinyl estradiol and 0.15 mg desogestrel pill vs. no estrogen or progesterone over 12 months	Spine and femoral neck BMD Z-scores significantly increased in the estrogen patch vs. the estrogen pill and no estrogen groups. Hip BMD Z-scores increased in the estrogen patch vs. the oral pill group
Singhal V, et al. The Journal of Clinical Endocrinology & Metabolism. 2019 (48).	73 oligo-amenorrheic females 14-25 years	100 µg 17β-estradiol transdermal patch twice weekly + cyclic 200 mg oral micronized progesterone vs. 30 µg ethinyl estradiol and 0.15 mg desogestrel pill vs. no estrogen or progesterone over 12 months	N-terminal propeptide of type 1 procollagen (P1NP), a marker of bone formation, decreased most in the pill group and this was associated with lower IGF-1 levels in the pill group vs. the other two groups, which did not have a decrease in IGF-1 levels; the pill group also demonstrated a marked increase in SHBG compared to the other two groups. The transdermal group had the greatest increases in estradiol levels and demonstrated decreases in sclerostin, preadipocyte factor-1 (Pref-1) and brain derived neurotrophic factor (BDNF). The increase in estradiol was directly related to increases in BMD.
Ackerman KE, et al. Journal of Bone and Mineral Research 2020 (125)	75 oligo-amenorrheic females 14-25 years	100 µg 17β-estradiol transdermal patch twice weekly + cyclic 200 mg oral micronized progesterone vs. 30 µg ethinyl estradiol and 0.15 mg desogestrel pill vs. no estrogen or progesterone over 12 months	Total and trabecular volumetric BMD, bone geometry and structural parameters improved in the estrogen patch vs. the estrogen pill group, particularly at the distal tibia.
			
***Other treatment options***			
Grinspoon S, et al. J Clin Endocrinol Metab. 2002 (126).	60 adult women with AN and osteopenia in their 3^rd^ decade of life	rhIGF-1 30 µg/kg SC BID + COC 35mcg ethinyl estradiol and 0.4 mg norethindrone vs. rhIGF-1 alone vs. COC alone vs. neither over 9 months	Combined therapy (rhIGF-1 plus COC) group had the greatest increase in BMD compared to the group that received neither.
Welt CK, *et al.* New England Journal of Medicine. 2004 (80).	14 women with FHA, 19-38 years	Metreleptin, recombinant human leptin, (n=8) vs. placebo (n=6) over 3 months	Treated group showed improved reproduction function and many resumed menses. Free triiodothyronine, free thyroxine, IGF-1, IGF binding protein 3, and bone formation markers (bone specific alkaline phosphatase and osteocalcin) increased in treated group. Treated group had a decrease in body weight and fat mass.
Golden NH, *et al.* The Journal of Clinical Endocrinology & Metabolism. 2005 (127).	32 adolescent girls with AN, mean age 16.9 years	Alendronate 10 mg daily vs. placebo over 1 year	Small but significant increase in BMD at the femoral neck, but not at the spine, in the alendronate vs. placebo groups after controlling for body weight changes over the study duration. Body weight was the best predictor of improved BMD
Chou SH, *et al.* Proceedings of the National Academy of Sciences. 2011 (128)	20 adult women with FHA 19-34 years	Metreleptin (n=11) vs. placebo (n=9) over 36 weeks	Improved menstrual function but no difference in spine, hip, radius or total BMD in the treated vs. placebo groups (though bone mineral content increased in the metreleptin group). Significant increase in a bone formation marker (osteocalcin). However, the treated group had a decrease in body fat.
Miller KK, et al. J Clin Endocrinol Metab. 2011 (129).	77 adult women with AN, in their 3^rd^ decade of life	Risedronate 35 mg weekly vs. low-dose transdermal testosterone vs. combination therapy vs. placebo over 12 months	Risedronate lead to increased posteroanterior spine, lateral spine and hip BMD compared to placebo. Testosterone administration was not associated with increased BMD but was associated with increased lean body mass
Divasta AD, et al. Metabolism. 2012 (130).	80 young women with AN 13-27 years	DHEA (50 mg daily) + COC (20 µg ethinyl estradiol + 0.1 mg levonorgestrel) (n=43) vs. placebo (n=37) over 18 months	DHEA + COC group had maintenance of spine and whole-body areal BMD Z-scores while placebo group had a decrease in areal BMD Z-scores over the study duration
Fazeli PK, et al. The Journal of Clinical Endocrinology & Metabolism. 2014 (131).	32 women with AN, mean age 47 years	Recombinant PTH (n=21) vs. placebo (n=11) over 6 months	Spine BMD improved in the recombinant PTH group compared to the placebo group
Divasta AD, et al. J Adolesc Health. 2019 (132).	70 adolescent girls with AN 11-17 years	Oral micronized DHEA (50 mg daily) + COC (20 µg ethinyl estradiol + 0.1 mg levonorgestrel) (n=35) vs. placebo (n=35) over 12 months	Reduction in BMD Z-scores in girls with open epiphysis and no change in girls with at least one closed epiphysis with combination therapy compared to placebo No effect of treatment on pQCT parameters at the tibia
Haines MS, et al. J Bone Miner Res. 2021 (133).	90 women with AN and low areal BMD Z-scores, 19-35 years	Sequential therapy with rhIGF-1 over 6 months followed by risedronate for 6 months (n=33) vs. 12 months risedronate (n=33) vs. placebo (n=16)	rhIGF-1+risedronate therapy was associated with greater spine areal and volumetric BMD than the placebo group and greater spine areal BMD than the other groups.
Haverinen A, et al. The Journal of Clinical Endocrinology & Metabolism. 2022 (134).	59 healthy women, 18-35 years	Estradiol valerate 2 mg + dienogest 2-3 mg (n=20) vs. ethinyl estradiol 30 µg + dienogest 2 mg (n=20) vs. dienogest 2mg (n=19) over 9 weeks	Lower levels of SHBG, and less pronounced FSH suppression leading to higher estradiol levels in the estradiol valerate vs. the ethinyl estradiol and dienogest groups.

Anorexia nervosa (AN); combined oral contraceptive (COC); dual energy x-ray absorptiometry (DEXA); functional hypothalamic amenorrhea (FHA); Bone mineral density (BMD); insulin-like growth factor (IGF); parathyroid hormone (PTH); Recombinant human IGF-1 (rhIGF-1); dehydroepiandrosterone (DHEA); peripheral quantitative computed tomography (pQCT).

The most important strategy to improve bone density in adolescent and adult women with FHA is normalization of menstrual function and recovery of weight (for those who are undernourished, underweight or have had recent weight loss). It is important to supplement vitamin D to maintain 25(OH) vitamin D levels above 30 ng/ml (135) and to recommend adequate calcium intake (1000-1500 mg daily). Clinicians should obtain a baseline BMD measurement by dual-energy X-ray absorptiometry (DXA) for any adolescent or woman with 6 or more months of amenorrhea, and even earlier in patients with a history of severe nutritional deficiency, other energy deficit state, and/or skeletal fragility (136).

Although estrogen deficiency is an important determinant of low BMD in FHA, many studies have shown lack of a protective effect of combined oral contraceptives (COCs) on bone (120–122, 137). A possible reason for the lack of efficacy of oral estrogen in increasing BMD is the suppression of IGF-1, a key osteoanabolic hormone (particularly during the adolescent years), by COCs because of hepatic first pass metabolism (123, 138). In contrast, transdermal 17-β estradiol, administered in replacement doses, is not IGF-1 suppressive (138, 139), and randomized clinical trials (47, 124) over 12 or 18 months have demonstrated that transdermal 17-β estradiol replacement is effective in increasing spine and hip BMD Z-scores in oligo-amenorrheic athletes and adolescents with AN, although catch-up is incomplete. This lack of complete catch-up is likely due to residual alterations that persist in other hormones that may impact bone that are not fixed by estrogen replacement. Importantly, the impact of estrogen replacement on fracture risk in women with FHA remains unclear. The Endocrine Society guidelines suggest short-term use of transdermal 17-β estradiol with cyclic oral progestin in adolescents and women with FHA who do not resume menses after a reasonable trial of nutritional, psychological, and/or modified exercise intervention (136).

The route of estrogen administration may have an impact on bone that extends beyond effects on IGF-1. A study that examined the impact of route of estrogen administration in oligo-amenorrheic athletes showed that transdermal estradiol replacement was associated with an increase in estradiol levels (associated with increases in bone density), and a decrease in factors that inhibit osteoblastic activity such as sclerostin, Pref-1, and brain-derived neurotrophic factor (BDNF). Further, while COCs led to a significant increase in sex hormone binding globulin levels with a decrease in levels of bioavailable gonadal steroids, this effect was not observed in the transdermal estrogen group (139). All these mechanisms may contribute to the efficacy of transdermal estrogen (but not COCs) in improving bone outcomes. A study examining effects of estradiol valerate versus ethinyl estradiol in oral contraceptive pills with the same progestin found a less pronounced FSH suppression in the estradiol valerate group, leading to higher estradiol levels and suggesting more positive effects of natural estradiol on bone mass (134).

Adolescent girls and adult women with AN and amenorrheic athletes have lower levels of testosterone than control groups. However, one study of transdermal testosterone given in replacement doses vs. placebo in adult women with AN was not associated with increases in BMD, despite an initial increase in bone formation markers (129).

Few studies have evaluated the use of anti-resorptive medications such as bisphosphonates and denosumab in FHA. One randomized controlled study of risedronate vs. placebo in adult women with AN reported small but significant increases in BMD (2-3%) at the spine and hip (129), while another study of alendronate vs. placebo in adolescents with AN reported a small increase at the femoral neck (but not at the spine) (127). When considering these drugs as a therapeutic strategy (particularly in women who have repeated fractures despite lifestyle intervention, optimization of calcium and vitamin D status, and estrogen replacement), caution needs to be exercised during the reproductive years given concerns regarding their long half-life. Data for denosumab are not available at this time in women with FHA.

Similar to the anti-resorptives, few studies have examined the impact of osteoanabolic drugs [such as teriparatide, recombinant PTH (rPTH), abaloparatide, romosozumab, recombinant leptin (metreleptin), rhIGF-1, and DHEA] on bone outcomes in FHA. A 6-month study of teriparatide vs. placebo in older pre-menopausal women with AN reported improvements in spine BMD (131); however, studies over a longer duration are currently lacking. There are also no studies that have reported on the impact of abaloparatide or romosozumab on bone outcomes in FHA.

Although a small 3-month study of metreleptin vs. placebo in adult women with FHA demonstrated improvement in menstrual function and increases in levels of IGF-1 and markers of bone formation with metreleptin, the medication led to subjective reductions in appetite and a significant decrease in body weight and fat mass (80). A subsequent small 9-month study similarly showed improved menstrual function and an increase in bone mineral content at the lumbar spine following metreleptin treatment. However, the group that received this drug had a significant decrease in body fat despite careful dose titration to prevent weight loss, an undesirable side effect in individuals with FHA (128).

Recombinant human IGF-1 given with a COC was demonstrated to increase spine and hip BMD in adult women with AN in a 9-month RCT in which the women were randomized to receive the combination regimen, rhIGF-1 alone, COC alone or neither (126), suggesting that administering rhIGF-1 in replacement doses may mitigate the IGF-1 suppressive effects of a COC. However, in a 12-month randomized controlled trial in adolescents with AN in which all received transdermal 17-β estradiol (given in replacement doses with cyclic oral progestin) with half being randomized to receive replacement doses of rhIGF-1 and half randomized to placebo, adding rhIGF-1 to transdermal 17-β estradiol did not lead to a further improvement in bone outcomes (140). In contrast, in a study in adults with AN, sequential therapy with rhIGF-1 for 6 months followed by risedronate for 6 months (vs. 12 months of risedronate or double placebo) led to greater increases in spine aBMD and vBMD than in the double placebo group, and greater increases in lateral spine aBMD than in both other groups (133).

Finally, one 18-month study demonstrated that a combination regimen of DHEA (50 mg daily) with a COC (vs. double placebo) led to a maintenance of BMD Z-scores at multiple sites compared to a decrease in these measures in the placebo group in young women with AN 13-27 years old (130). However, a subsequent study of this combination regimen vs. placebo in younger girls with AN 11-17 years old reported a reduction in spine and whole-body BMD Z-scores with the combination regimen in the younger girls with open epiphyses, and no change in these BMD measures in older girls with open epiphyses (132).

Based on these and other studies, current guidelines caution against using denosumab, metreleptin and androgens to improve bone outcomes in FHA. In rare adult FHA cases, the guidelines suggest short-term use of teriparatide as an option in patients with delayed fracture healing and very low BMD (136). Given that recent studies have demonstrated that bisphosphonates improve bone outcomes in adults with AN (133) this may be a consideration in those women who continue to have fractures despite attention to caloric intake, a reduction in exercise activity, and optimization of vitamin D, calcium, and estrogen status.

## Neuropsychiatric outcomes

Adequate estrogen status is essential for optimal cognitive function and may also impact mood and emotion (141, 142).

**Cognitive Function:** Hypoestrogenism has deleterious effects on verbal memory and executive function (specifically cognitive flexibility) in oligo-amenorrheic athletes compared to eumenorrheic ones and/or non-athletes (143). Other studies have also demonstrated cognitive dysfunction in adolescent and adult women with FHA (144, 145), with an improvement in these measures with menstrual resumption or estrogen administration (144). Importantly, estrogen replacement as the transdermal 17-β estradiol patch with cyclic oral progesterone given for a 6-month period improved both verbal memory and cognitive flexibility in oligo-amenorrheic athletes compared to a no-estrogen group (with a COC group demonstrating intermediate effects) (143).

**Emotion and Mood:** Women with FHA have significantly higher depression and anxiety scores compared to healthy controls (146). Studies reported that depression and anxiety are common in women with FHA, suggesting a role for estrogen in mediating these effects (146–149). Another study reported that administration of transdermal estradiol reduced trait anxiety in girls with AN and prevented the increase in state anxiety observed with weight gain over time than in those who received placebo (150).

**Eating Behaviors and Attitudes:** Women with FHA exhibit more dysfunctional attitudes such as perfectionistic behavior and extra attention to peoples’ judgments and have great difficulty coping with daily stress in comparison with eumenorrheic women (151, 152). Additionally, women with FHA report greater internal feelings of insecurity, inadequacy, and lack of control over their lives (146). One study in athletes and non-athletes reported greater cognitive restraint, drive for thinness, feelings of ineffectiveness and greater interoceptive awareness in oligo-amenorrheic athletes compared to eumenorrheic athletes and non-athletes (153). A subsequent study showed a significant improvement in drive for thinness and body dissatisfaction scores, and a reduction in uncontrolled eating after 12 months of treatment with transdermal estradiol with cyclic progesterone (154) (not observed in those who received COCs). Further, transdermal estradiol replacement in adolescent girls with AN has been demonstrated to prevent the increase in body dissatisfaction that occurs with weight gain over time in those who remain hypoestrogenic (150).

**Hormonal Correlates of Neuropsychiatric Outcomes:** Estrogen has an influence on many areas of the brain including to the hypothalamus, cerebellum, nigrostriatal and mesolimbic system, amygdala, hippocampus, cerebral cortex, and brainstem (155). Estrogen also modulates many neurotransmitters including serotonin, acetylcholine, dopamine, and norepinephrine (156). Although hypoestrogenemia plays a major role in the neurocognitive impairment in FHA, hypercortisolemia due to HPA dysregulation and fluctuations in neuropeptides and neurotransmitters can work synergistically to promote the neuropsychiatric disturbances in this condition. One study showed that amenorrheic women present greater increases in heart rate, systolic and diastolic blood pressure, and serum cortisol levels in response to neuropsychological stress exposure than eumenorrheic women (151). In another study of 21 healthy controls, 18 amenorrheic women with AN, and 13 normal-weight women with FHA, cortisol levels showed a strong correlation with anxiety and depressive symptoms (30). Lower levels of gonadal hormones, oxytocin, and leptin, and higher levels of cortisol and PYY have been implicated in eating disorder psychopathology and symptoms of anxiety and depression in AN (31, 32, 108, 157).

The relationship between psychological stress and FHA is bidirectional, as stress can trigger the suppression of the HPO axis and, conversely, low levels of estrogen greatly impact the neuropsychological status, thus creating a vicious cycle. Therefore, psychological support is essential to break the cycle. The Endocrine Society Clinical Practice Guidelines (136) suggest psychological treatment such as cognitive behavioral therapy (CBT) to improve the ability to cope with psychological stressors. In a study, eight women with FHA were randomized to CBT and eight to observation for 20 weeks. Among women who received CBT, most (six of eight) achieved ovulatory recovery compared to only one of eight in the observation group (116). In another study, CBT lowered cortisol levels, and increased leptin and TSH levels in women with FHA (115). The long-term impact of CBT in amenorrheic women needs to be studied.

## Conclusion

**Figure 4:**
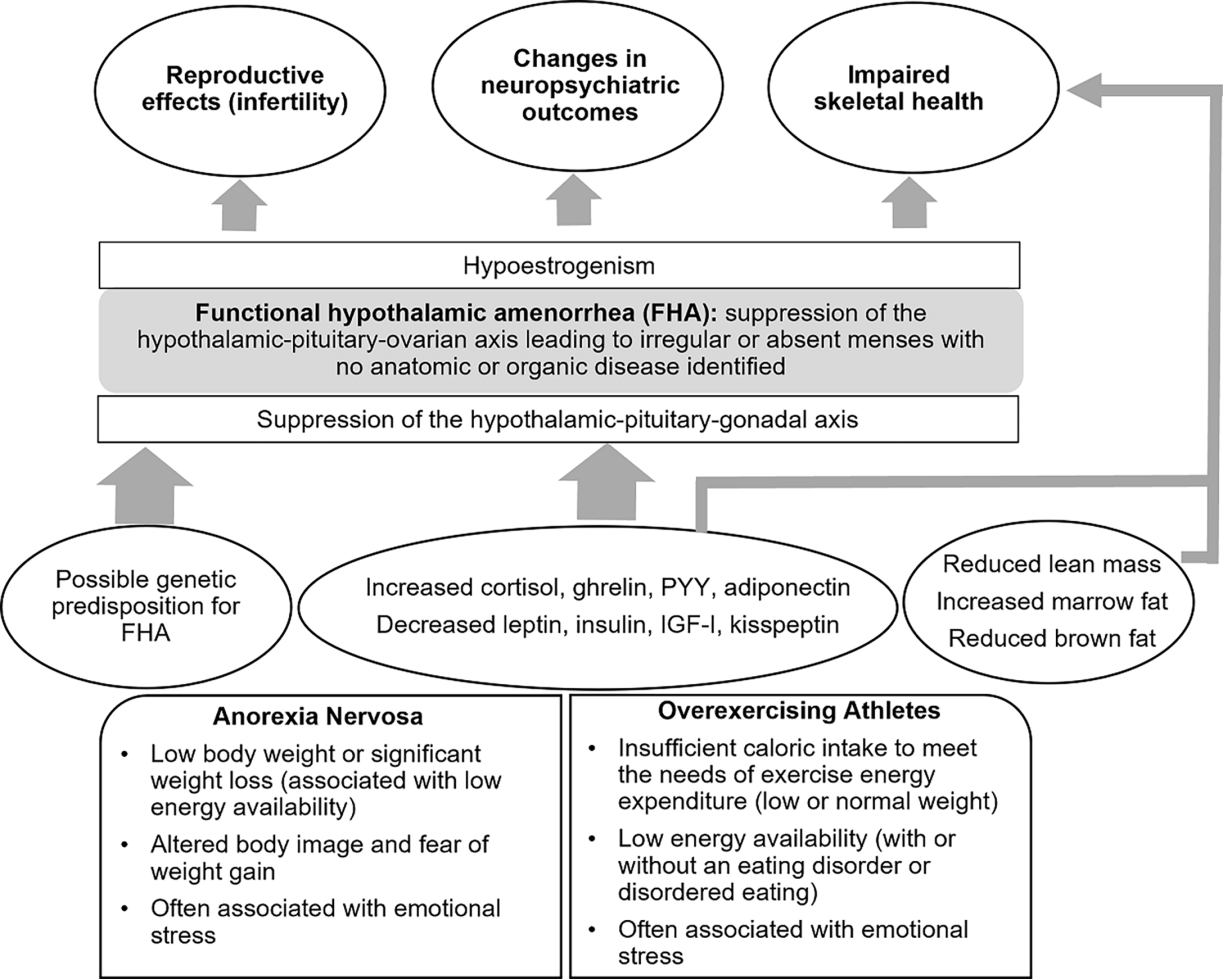
Anorexia Nervosa and the Female Athlete Triad.

FHA from AN, low energy availability in athletes or chronic stress is a frequent cause of oligo-amenorrhea in young women and can go undiagnosed for long periods of time. FHA results from disruption of the HPO axis consequent to other endocrine changes and possibly a genetic predisposition, with an impact on reproductive, neuropsychiatric and skeletal health (summarized in **Figure 4**). Early recognition of patients at risk of developing FHA is very important due to the long-term consequences of low energy availability on the reproductive system, and the impact of low energy availability and hypoestrogenism on bone and neurocognitive outcomes, particularly during the critical adolescent and young adult years when skeletal and neurological systems are maturing. Treatment aims to optimize energy availability with restoration of gonadal function and generally requires a multidisciplinary team. Transdermal estrogen therapy is a proven useful tool in those women who do not respond to nutritional, psychological, and/or modified exercise intervention, and has beneficial effects on bone accrual, as well as neuropsychiatric outcomes.

## Author contributions

All authors listed have made a substantial, direct, and intellectual contribution to the work and approved it for publication.

## Conflict of interest

The authors declare that the research was conducted in the absence of any commercial or financial relationships that could be construed as a potential conflict of interest.

## Publisher’s note

All claims expressed in this article are solely those of the authors and do not necessarily represent those of their affiliated organizations, or those of the publisher, the editors and the reviewers. Any product that may be evaluated in this article, or claim that may be made by its manufacturer, is not guaranteed or endorsed by the publisher.
